# A CBCT based analysis of the correlation between volumetric morphology of the frontal sinuses and the facial growth pattern in caucasian subjects. A cross-sectional study

**DOI:** 10.1186/s13005-022-00308-3

**Published:** 2022-02-02

**Authors:** Andrea Abate, Francesca Gaffuri, Valentina Lanteri, Andrea Fama, Alessandro Ugolini, Laura Mannina, Cinzia Maspero

**Affiliations:** 1 Department of Biomedical Surgical and Dental Sciences, University of Milan, 20142 Milan, Italy; 2 Ospedale Maggiore Policlinico, Fondazione IRCCS Cà Granda, 20142 Milan, Italy; 3 Department of Sciences Integrated Surgical and Diagnostic, University of Genova, Genova, Italy

**Keywords:** Frontal sinuses, Volumetric analysis, CBCT, Facial growth pattern

## Abstract

**Background:**

The aim of this study was to evaluate the relationship between frontal sinus shape and facial growth pattern.

**Methods:**

The three-dimensional examination was carried out by means of 80 CBCT scans selected from a sample of 1247 records of patients treated, for different reason, at the Department of Biomedical Surgical and Dental Sciences at University of Milan, Fondazione IRCCS Ca’ Granda, Ospedale Maggiore Policlinico Milan. The sample (age ranges between 12 and 40 years) was divided according to gender and age in four groups (12-17, 18-20, 21-30, 31-40). Left and right frontal sinus volume (VOL), surface (SUP) and linear maximum width (XMAX), depth (ZMAX) and height (YMAX) were calculated using Mimics Research 17.0 (Materialise N.V., Leuven, Belgium). Cephalometric analysis has been performed for all subjects to categorize the patients depending on their facial growth pattern. Univariate and multivariate regression analysis were performed to investigate any association of frontal sinuses measurements (height, width, depth, volume and surface) and cephalometric variables. P value < 0.05 was considered statistically significant.

**Results:**

A total of 160 frontal sinuses were measures in 80 patients: 40 men and 40 women, average age of 23.5 ±14.6. Globally the frontal sinuses had the following average dimensions: volumes of 9055.8 ± 6505 mm^3^ and surfaces of 3820.3 ± 2125 mm^2^. The statistical analysis showed that frontal sinus volume was statistically significant (*p*=0.003) greater for male (11,425 mm^3^) than female (6597.5 mm^3^). Similarly, the surface showed to be greater in men than in women (*p*=0.005). No correlation between age and frontal sinuses characteristics has been found. A statistically significant (*p*<0.05) increase of frontal sinus depth, surface and volume was correlated with SNB angle. In addition, frontal sinus volume increased in subjects with greater anterior skeletal dimension values and with a superior length of the cranial base. Furthermore, a decrease of ANB has been found related to an increase in frontal sinus volume (*p*=0.04).

**Conclusions:**

The present study showed a correlation between frontal sinuses dimensions and craniofacial aspects, despite the inter-individual variability of their morphology. The results suggested that young adults in whom the frontal sinuses have reached their maximum size, while vertical growth continues, a larger frontal sinus may be associated with future vertical growth.

**Supplementary Information:**

The online version contains supplementary material available at 10.1186/s13005-022-00308-3.

## Background

The study of morphology and development of the frontal sinuses has been of interest in the anthropological and forensic fields for the determination and identification of cadavers [1–3]. Several studies were performed to investigate the correlation between frontal sinus morphology, age, and sex in the identification of humans [4, 5]. The frontal sinus originates from the ethmoidal cells, which migrate into the frontal bone at the end of the first year of life. Unlike the other sinuses, the frontal sinuses are not visible at birth and may be detectable radiographically from 4 to 6 years of age according to some sources [6, 7]. These results are not in agreement with a study by Dolan [8]. In fact, the author reports that the frontal sinuses are detectable at the age of 8.

The volume of the frontal sinuses increases until the age of twenty, following the growth of the craniofacial structures [9, 10].

Many studies have shown that the growth of the maxilla and nasal cavities is closely related to the development of the sinuses, and these structures ultimately determine the morphology of the face [11, 12]. Rae et al. suggested many possible functions, including respiratory function, thermoregulation, protection from trauma, and to decrease skull weight [13].

Studies in twins state that the shape of the sinuses is primarily determined by genetics [1, 14]. However, environmental factors, trauma, allergies, acquired conditions, nutrition, and medications can affect sinus development [15]. Koertvelyessey has reported a correlation between cold climates and the degree of pneumatization of the frontal sinus [16]. Therefore, genotype and growth of maxillofacial structures are two of the main factors that can influence frontal sinus size [14].

Witzel et al. reported that the sinuses developed in response to biomechanical needs of the skull structure [17]. The amount and direction of chewing forces are the primary factors inducing mechanical forces in the maxillo-facial structure. These processes influence the degree of pneumatization of the sinuses. The distribution of masticatory forces through the human skull has been shown by several studies performed with structural analysis using finite elements [17, 18].

These high-intensity forces are distributed from the dental arches, along the medial periphery of the orbits. The presence of a septum in the frontal sinus seems to be a consequence of stress distribution on the midline, suggesting that these masticatory forces reach the frontal sinus.

Furthermore, Throckmorton et al. [19] confirmed that orthognathic surgery with the achievement of a more harmonious maxillo-mandibular relationship can lead to a more favorable transfer of stresses along the facial skeleton.

Subsequently, Prado et al. demonstrated that 6 months after correction of a Class II open-bite malocclusion, achieved by maxillo-mandibular advancement with counterclockwise rotation of the occlusal plane, a reduction in frontal sinus size was observed. The authors attributed this change to an adaptation to the stresses induced by a more favorable occlusion [20]. Some recent studies have reported having made a 3D evaluation and the other paranasal sinuses and the rhynopharynx for surgical and medical purposes [21, 22]. Some recent publications have stressed the possibility of studying frontal sinuses by CBCT with high reliability [7, 23–25]. This technique provides very good opportunities for studying these anatomical structures with a three-dimensional (3D) approach and great precision in all measurements [6, 23].

Based on a literature research performed on Pubmed, Scopus and EbSco by the authors it was evinced that there are a limited number of papers [26, 27] investigating the relationship between frontal sinus size and cephalometric characteristics performed with three-dimensional imaging technique. Most of the researches have been performed using bidimensional radiographs such as lateral cephalograms and posteroanterior radiogram [28, 29].

Thus, the primary aim of the present volumetric analysis was to investigate the correlation between the three-dimensional morphology of the frontal sinuses and facial growth pattern using CBCT scans and the secondary aim was to compare volume, surface and linear measurements of frontal sinuses within the three age groups and between genders.

## Methods

### Selection of the patients

A cross-sectional study was conducted on CBCT images of patients who attended the Department of Biomedical Surgical and Dental Sciences at University of Milan, Fondazione IRCCS Ca’ Granda, Ospedale Maggiore Policlinico Milan, during the period of 2015-2021.

The study protocol was approved by the Ethical Committee of the Fondazione IRCCS Ca’ Granda, Ospedale Maggiore, Milan, Italy (protocol no. 573/15) and all the procedures carried out during this study complied with the ethical standards of the institutional and research committee and with the declaration of Helsinki of 1975 and revised in Tokyo 2004. All the patients or their parents signed an informed consent to permit us to use their medical records for research investigations in anonymous form.

### Inclusion and exclusion criteria

From 1247 patients, whose CBCT images were taken for diagnosis and treatment plan of craniofacial, skeletal, or pathological abnormalities, male and female patients meeting the following inclusion criteria were selected: (1) patients over the age of 12 years (mean age of 23.5).

Exclusion criteria were:(1) individuals with respiratory disease;(2) syndromes including craniofacial bones;(3) clefts, hemifacial microsomia; (4) patients with endocrine and metabolic pathologies;(5) lesions with bilateral clinical involvement, relapses of previously diagnosed and/or treated lesions; (6) lesions which affect the maxillary sinus due to locoregional spread but which originate in a different location; (7) lesions with odontogenic origin and malignant neoplasms; (8) presence of infections or pathological signs affecting the frontal sinuses; (9) previews orthognathic surgery; (10) dental anomalies.

A total of 80 subjects with a CBCT scan were selected and divided according to their age into four age groups of 20 individuals: group one 12-17 (10 males and 10 females), group two 18-20 (10 males and 10 females), group three 21-30 (10 males and 10 females), group four 31-40 (10 males and 10 females).

### CBCT analysis and three-dimensional reconstruction of the frontal sinuses 

The 3D images were obtained using an I-CAT FLX (Imaging Sciences International, Hatfield, PA, USA) configured for the same exposure parameters for the patients exanimated. The scanning parameters were configured as follow: 360_ rotation, 300 frames, 120 kV[p], 5 mA, 3.7 s, voxel size 0.4 mm, field of view (FOV) 16 mm x 8 mm/16 mm x 11 mm, to minimize radiation exposure.

All records were investigated by means of Mimics Research 17.0 (Materialise N.V., Leuven, Belgium). Reconstruction of the coronal (x–z), sagittal (y–z) and transversal (x–y) planes were analyzed. Air has the lowest values of HU (Hounsfield Unit), while bone has the highest one. A specific threshold limits of -1024 HU (minimum) and -526 HU (maximum) were applied for the sinus volume calculation as suggested by previous studies [30, 31]. The line drawn parallel to the Frankfort horizontal plane and passing through the tightest area of the sinus before its connection with the nose and the lower airway was chosen as the baseline of the sinus as a reference limit, to determine the whole volume of the sinuses which sometimes could not be significantly isolated if the baseline was drawn in an area above.

**Fig. 1:**
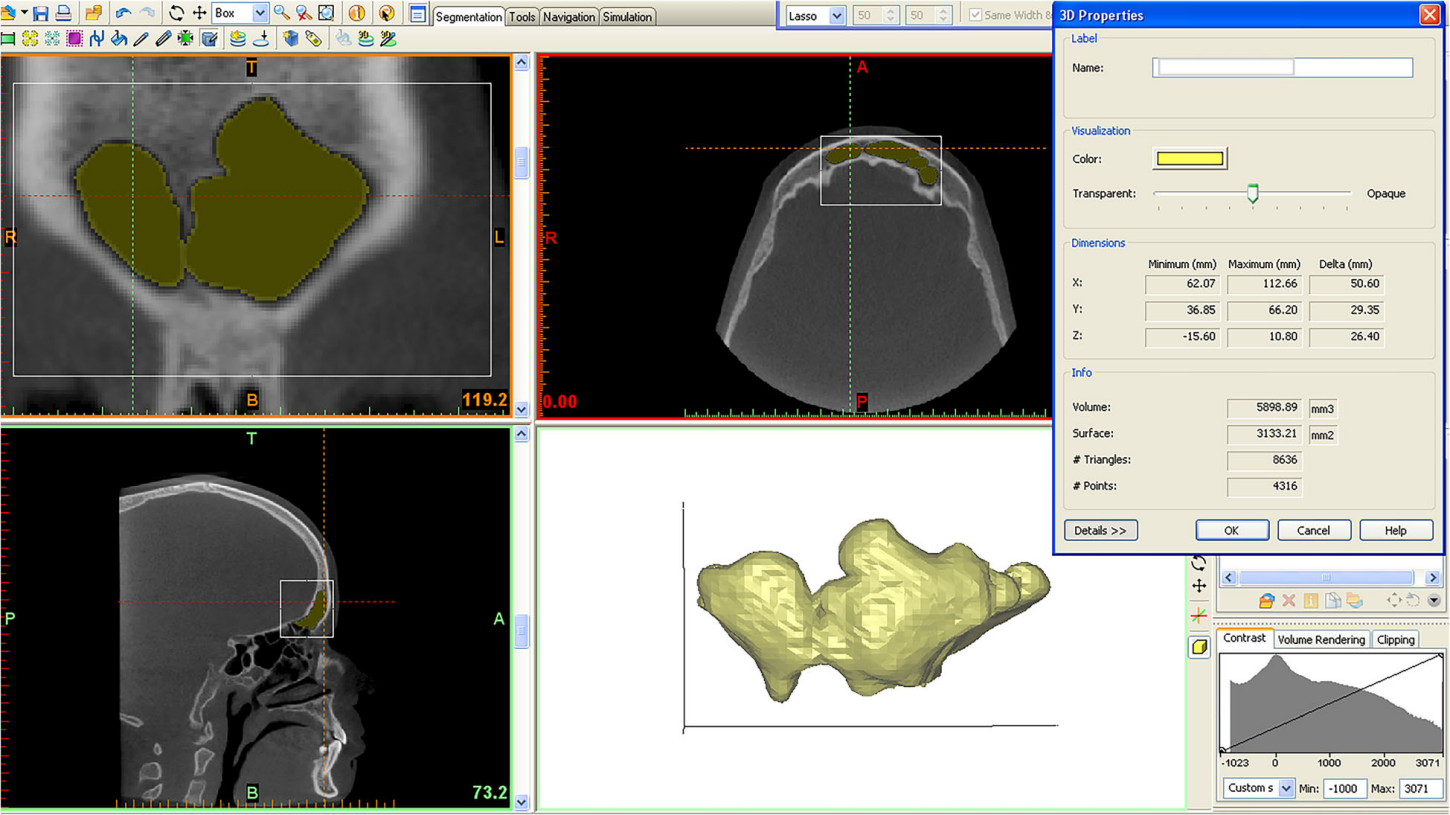
Three-dimensional reconstruction of the frontal sinus with the relative 3D characteristics extrapolated using Mimics Research 0 (Materialise N.V., Leuven, Belgium)

Frontal sinus volumes were calculated using the software’s “calculate 3D” tool. After segmentation, the three-dimensional volumetric structure of the frontal sinus volume of each patient was calculated separately (Fig. 1). Each frontal sinus was cropped using the software’s tool called “edit masks” along the borders and the “region growing” tool was used to split the segmentation created by thresholding into several objects, finally the smooth function was used to remove floating pixels.

The software Mimics Research automatically calculated: volume (VOL) in mm^3^, total surface (SUP) in mm^2^ and linear maximum width (XMAX), height (YMAX) and depth (ZMAX) in mm (Fig. 1).

**Fig. 2:**
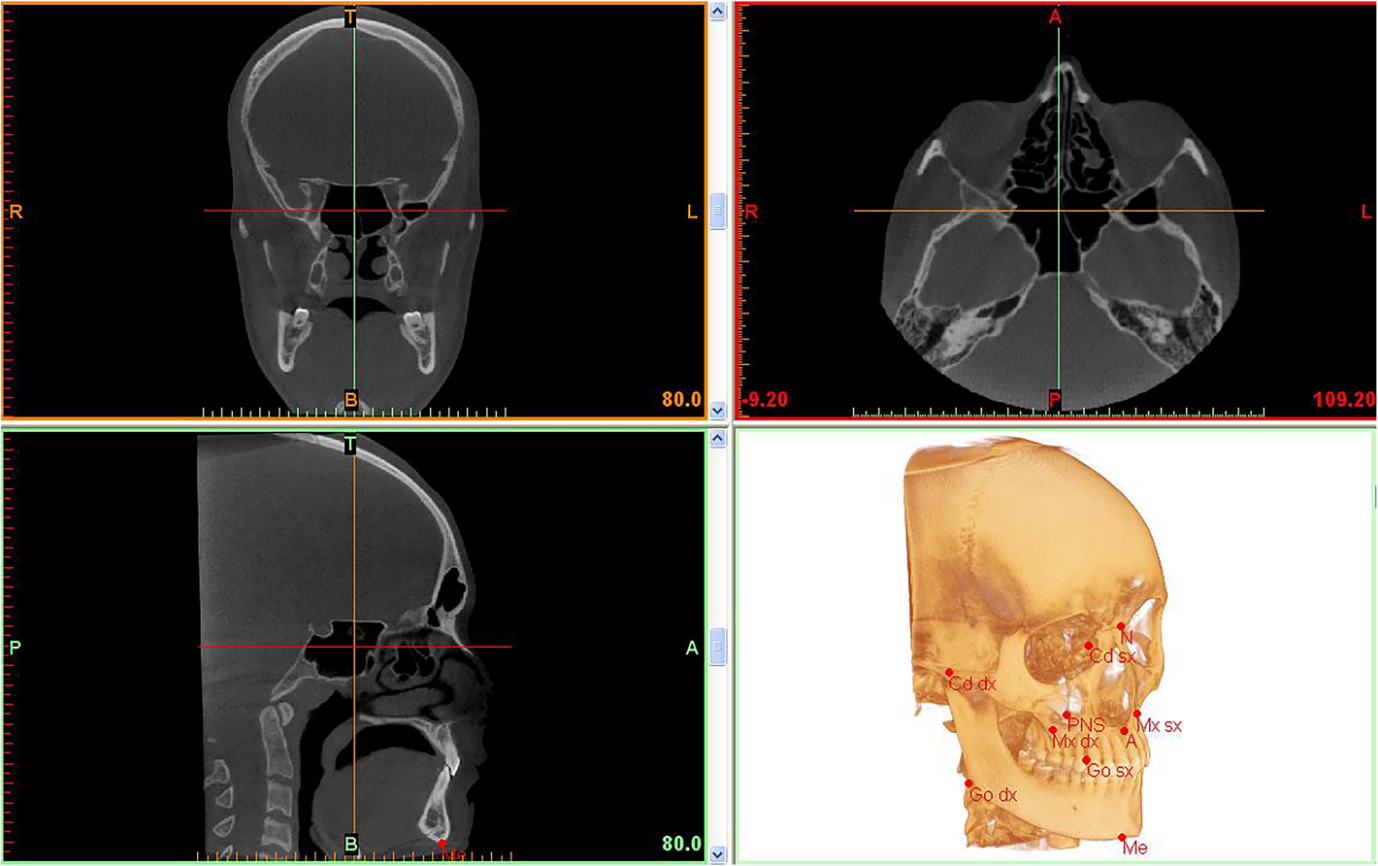
3D cephalometric analysis performed on Mimics Research (Materialise N.V., Leuven, Belgium)

Cephalometries tracings have been performed for each subject to evaluate the association between craniofacial features and frontal sinus characteristics, all variables detected are listed in Table 1. Figure 2 summarized the cephalometric protocols adopted in the present study [32, 33].

**Table 1:** Cephalometric variables investigated in the present study

Variables	Definition
SNA	the angle formed between points S, N, and A, indicating the anteroposterior projection of the maxilla
SNB	the angle formed between points S, N, and B, indicating the anteroposterior projection of the mandible
ANB	the angle formed between points A, N, and B, indicating the sagittal intermaxillary relationship
SN (Anterior cranial fossa length)	the distance between the sella point (S) and nasion point (N)
AH (Total anterior facial height)	the distance between the nasion point (N) and the menton point (Me)
AHU (Upper anterior facial height)	the distance between the nasion point (N) and the anterior nasal spine point (ANS)
AHL (Lower anterior facial height)	the distance between the anterior nasal spine (ANS) and menton point (Me)
DIV (Intermaxillary angle)	the angle between the bispinal plane (ANS-PNS) and the mandibular plane (Go- Me)

The variation of frontal sinuses measurements was investigated in relation to sex, age and cephalometric parameters.

### Statistical analysis

A priori sample size was computed using G*Power (version 3.1.9, http://www.psychologie.hhu.de/arbeitsgruppen/allgemeine-psychologie-und-arbeitspsychologie/gpower.html) on 40 subjects (10 for each age group); power calculation analysis was calculated analyzing the average values of volume of frontal sinus for each age group and the common standard deviation. The following data were used to calculate sample size: age 12-17 = 8285 mm^3^, 18-20= 9671 mm^3^, 21-30=14,810 mm^3^, 31-40=12,932 mm^3^; group size= 10; σ within each group = 4036 mm^3^; α = 0.05; with a beta error level of 20%. The analysis revealed that 32 subjects (8 in each group) were necessary to perform the study. However, according to the inclusion criteria, the authors included 20 patients per group, to increase the robustness of the results.

The software Stata 16 (StataCorp. 2019) was used for the statistical analysis. Dimensional variations of the volume, surface and linear maximum width, height and depth of the frontal sinus in the four age groups and between male and female were analyzed.

Descriptive statistics were performed for variables VOL, SUP, XMAX, ZMAX and YMAX.

The Kolmogorov–Smirnov test was used to assess whether the data was normally distributed. The statistical distribution of the quantitative measures was found not to be Gaussian. The variables were analyzed together using random-intercept linear regression models. Univariate and multivariate regression analysis were performed to investigate any association of frontal sinuses measurements (volume, surface, height, width and depth) and cephalometric variables. To take into account intra-subject correlations, we fitted random-intercept linear regression models to calculate slopes, 95% confidence intervals (CI) and p-values. We performed crude, adjusted (for gender and age), and stratified (by gender) analyses.

To test intra-observer reliability, a random sample of 30 CBCT scans were each re-examined twice, at two-week.

intervals by the first investigators. Assessment of inter-observer reliability was carried out by a second observers, that re-evaluated 30 randomly selected CBCT scans. Intra- and inter-observer reliability was studied with the intra-class correlation coefficient (ICC). whereas the Dahlberg’s formula was used for the evaluation of the method error.

P value < 0.05 was considered statistically significant.

## Results

A total of 160 frontal sinuses were measures in 80 patients (40 men and 40 women, with an age range from 12 to 40 and an average age of 23.5 (SD= 14.6). Descriptive statistics of the variables considered is summarized in Table 2.

**Table 2:** Descriptive statistics for the frontal sinus measurements

	Age 12-17		Age 18-20		Age 20-30		Age 30-40	
Variables	Mean	SD	Mean	SD	Mean	SD	Mean	SD
VOL	740.60	3758.66	7659.13	5032.60	10017.6	7836.19	11159.2	8144.80
SUP	3156.36	1232.57	3468.60	1667.38	4223.68	2443.42	4466.15	2736.60
XMAX	47.75	16.84	48.55	17.22	55.99	19.35	56.89	21.70
ZMAX	29.84	7.76	31.62	9.78	32.07	8.15	35.32	12.62
YMAX	23.93	5.27	26.28	4.47	27.19	9.75	25.60	6.97

The reliability of the method segmentation tested with the intra-observer and inter-observer reliability demonstrated high range of agreement: average (± SD, range) ICC were respectively: 0.962 (±0.042, 0.949–0.990) and 0.952 (± 0.019, 0.924–0.986).

Dahlberg’s formula showed that the random error for sinus volume, surface and linear measurements was respectively 512 mm^2^ for the volume, 98 mm^2^ for the surface and about 0.59 mm for the linear measurements.

### Comparisons of volume and surface and linear measurements of frontal sinuses within the age groups between genders

**Fig. 3:**
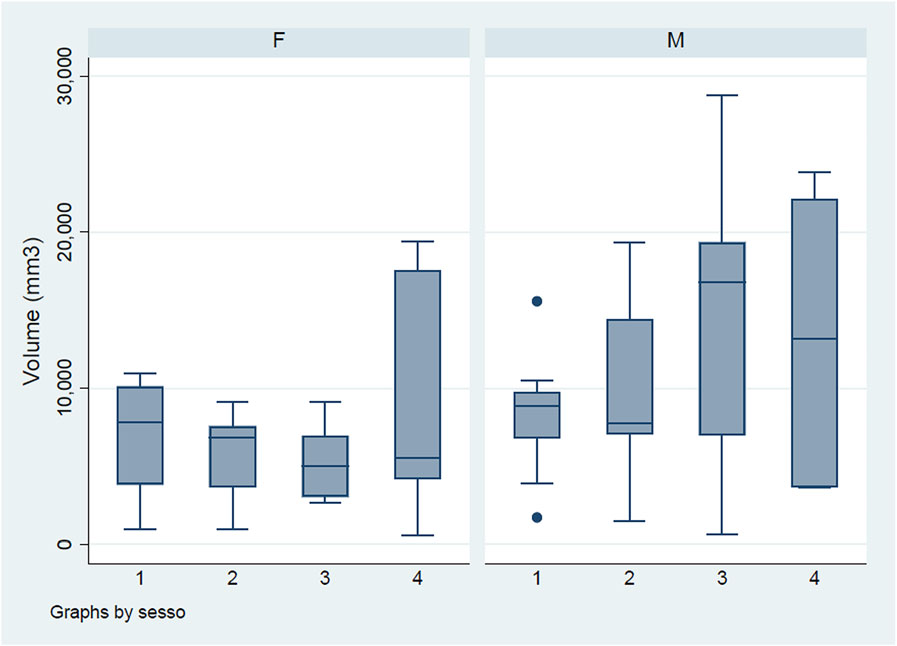
Average volume of the frontal sinus by sex in the sample

The volume of the frontal sinus is on average in male subjects (11,425 mm3), compared to female subjects (6624.5 mm3), with an evident statistical significance (*p* = 0.003). The same is not true for age, since although there is an increase in volume with age, however this is not statistically significant. Similarly, the surface area appears to be greater in men than in women (*p* = 0.005), but it does not increase in a statistically significant manner with age (Fig. 3).

Concerning the linear measurements, a difference in XMAX (width) was observed between males and females but with no statistical significance (*p*=0.06). Even the tendential increase of XMAX recorded with increasing age was not statistically significant.

A statistically significant difference was found between males and females for YMAX: a mean of 28.1mm for male versus 23.4 mm for females (*p* = 0.008). No significant differences in YMAX were detected between the age groups.

For the ZMAX parameter, no statistical significance was found associated to age or gender, although an increasing value of ZAMAX was correlated with increasing of age in males compared to females.

### Correlations between cephalometric analysis and frontal sinuses dimensions

Linear regression analysis did not reveal any statistically significant association between the skeletal class of subjects and their frontal sinus’s parameters. The results of the univariate and multiple regression analysis are summarized in Tables 3 and 4.

**Table 3:** Linear regression model considering the relationship between Frontal sinus and the selected cephalometric variables.

*Univariate regression model*										
	**VOL**		**SUP**		**XMAX**		**YMAX**		**ZMAX**	
	slope (95% CI)	p	slope (95% CI)	p	slope (95% CI)	p	slope (95% CI)	p	slope (95% CI)	p
SNA	1.42 (-3.68, 6.52)	0.60	1.40 (-2.38, 5.21)	0.40	0.46 (-0.60, 1.50)	0.40	1.50 (0.00, 3.00)	**0.05**	0.07 (-0.50, 0.60)	0.80
SNB	3.79 (0.05, 7.54)	**0.04**	2.74 (-0.05, 5.53)	**0.05**	0.70 (-0.70, 1.40)	0.10	1.41 (0.27, 2.54)	**0.01**	0.20 (-0.20, 0.70)	0.20
ANB	-4.88 (-9.67, -0.10)	**0.04**	-3.17 (-6.75, 0.42)	0.08	-0.70 (-1.70, 0.30)	0.10	-0.89 (-2.38, 0.60)	0.24	-0.30 (-0.88, 0.21)	0.23
DIV	-0.52 (-4.17, 3.13)	0.78	0.17 (-2.55, 2.89)	0.90	0.20 (-0.53, 0.94)	0.59	0.13 (-0.99, 1.24)	0.82	0.11 (-0.30, 0.52)	0.61
AH	4.18 (2.38, 5.98)	**0.00**	3.22 (1.89, 4.55)	**0.00**	0.84 (0.48, 1.20)	**0.00**	1.09 (0.51, 1.66)	**0.00**	0.46 (0.26, 0.66)	**0.00**
AHL	8.47 (3.91, 13.03)	**0.00**	6.64 (3.28, 10.00)	**0.00**	1.82 (0.92, 2.73)	**0.00**	1.92 (0.47, 3.37)	**0.01**	1.10 (0.61, 1.60)	**0.00**
AHU	3.55 (1.17, 5.93)	**0.00**	2.80 (1.04, 4.56)	**0.00**	0.69 (0.21, 1.17)	**0.01**	1.04 (0.31, 1.78)	**0.01**	0.40 (0.13, 0.67)	**0.00**
SN	6.01 (2.12, 9.91)	**0.00**	4.01 (1.08, 6.95)	**0.01**	0.87 (0.06, 1.68)	**0.03**	1.95 (0.76, 3.13)	**0.00**	0.55 (0.11, 1.00)	**0.02**

**Bold**: statistically significant difference.

**Table 4:** Multiple regression model analyzing the relationship between Frontal sinus characteristic and cephalometric parameters adjusted for age and gender

*Multiple regression model*										
	VOL		SUP		XMAX		YMAX		ZMAX	
	slope (95% CI)	p	slope (95% CI)	p	slope (95% CI)	p	slope (95% CI)	p	slope (95% CI)	p
SNA	0.74 (-4.2, 5.6)	0.80	0.97 (-2.72, 4.65)	0.60	0.36 (-0.60, 1.40)	0.50	1.31 (-0.10, 2.80)	0.08	0.02 (-0.5, 0.6)	0.90
SNB	2.06 (-1.86, 5.99)	0.30	1.60 (-1.35, 4.56)	0.28	0.40 (-0.30, 1.20)	0.30	0.89 (-0.3, 2.1)	0.10	0.10 (-0.40, 0.50)	0.70
ANB	-2.60 (-7.64, 2.44)	0.31	-1.60 (-5.40, 2.19)	0.40	-0.30 (-1.40, 0.70)	0.09	0.07 (-1.62, 1.48)	0.92	-0.10 (-0.70, 0,45)	0.67
DIV	0.98 (-2.66, 4.62)	0.59	1.22 (-1.50, 3.95)	0.37	0.44 (-0.29, 1.18)	0.23	0.64 (-0.46, 1.75)	0.25	0.24 (-0.17, 0.65)	0.25
AH	3.72 (1.56, 5.88)	**0.01**	3.08 (1.48, 4.68)	**0.00**	0.86 (0.43, 1.29)	**0.00**	0.84 (0.15, 1.53)	**0.01**	0.47 (0.23, 0.71)	**0.00**
AHL	6.56 (1.36, 11.77)	**0.01**	5.64 (1.78, 9.50)	**0.00**	1.67 (0.64, 2.71)	**0.00**	1.12 (-0.52, 2,0.76)	0.18	1.05 (0.49, 1.62)	**0.00**
AHU	2.53 (2.58, 5.10)	**0.05**	2.18 (0.29, 4.07)	**0.02**	0.55 (0.04, 1.06)	**0.03**	0.73 (- 0.05, 1.50)	0.07	0.32 (0.04, 0.61)	**0.03**
SN	4.09 (-0.94, 9.11)	0.11	2.71 (-1.09, 6.50)	0.16	0.59 (-0.44, 1.62)	0.26	1.30 (-0.24,2.83)	0.09	0.46 (-0.12, 1.03)	0.12

**Bold:** statistically significant difference.

The volume of frontal sinus has a statistically significant decreasing of 4.8% as the ANB value increase. Furthermore, the volume (4.2%) and the surface (3.2%) increasing with statistically significant as the skeletal anterior height increases as reported in Table 3.

**Fig. 4:**
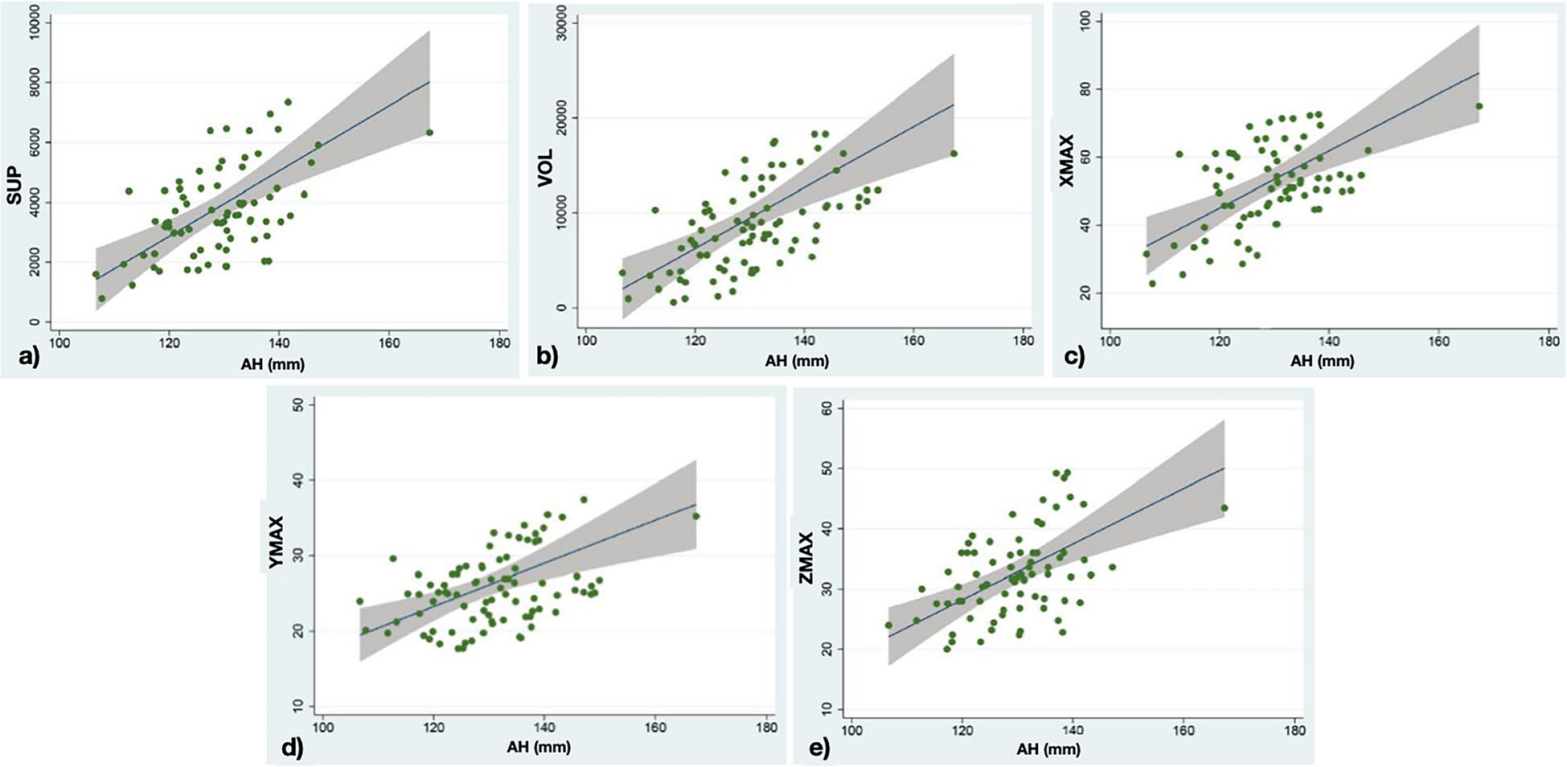
**A** Linear regression between the surface dimensions of the frontal sinus (sup), in the ordinate, and the total anterior skeletal vertical dimension (AH), in the abscissa. **B** Linear regression between the volume quantities of the frontal sinus (vol), in the ordinate, and the total anterior skeletal vertical dimension (AH), in the abscissa. **C** Linear regression between the width of the frontal sinus (xmax), in the ordinate, and the total anterior skeletal vertical dimension (AH), in the abscissa. **D** Linear regression between the depth of the frontal sinus (ymax), in the ordinate, and the total anterior skeletal vertical dimension (AH), in the abscissa. **E** Linear regression between the height of the frontal sinus (zmax), in ordinate, and total anterior skeletal vertical dimension (AH), in abscissa

A statistically significant increasing has been found for XMAX (0,84 mm), YMAX (1,1%), ZMAX (4,62 mm), VOL (4,2%) and SURF (3,2%) of the frontal sinus, corresponding to the increase of the anterior vertical dimension (Fig. 4).

The univariate regression analysis showed the relationship between the upper anterior vertical skeletal dimension and the parameters XMAX, YMAX, ZMAX, volume and surface of the frontal sinus as showed in Fig. 4.

**Fig. 5:**
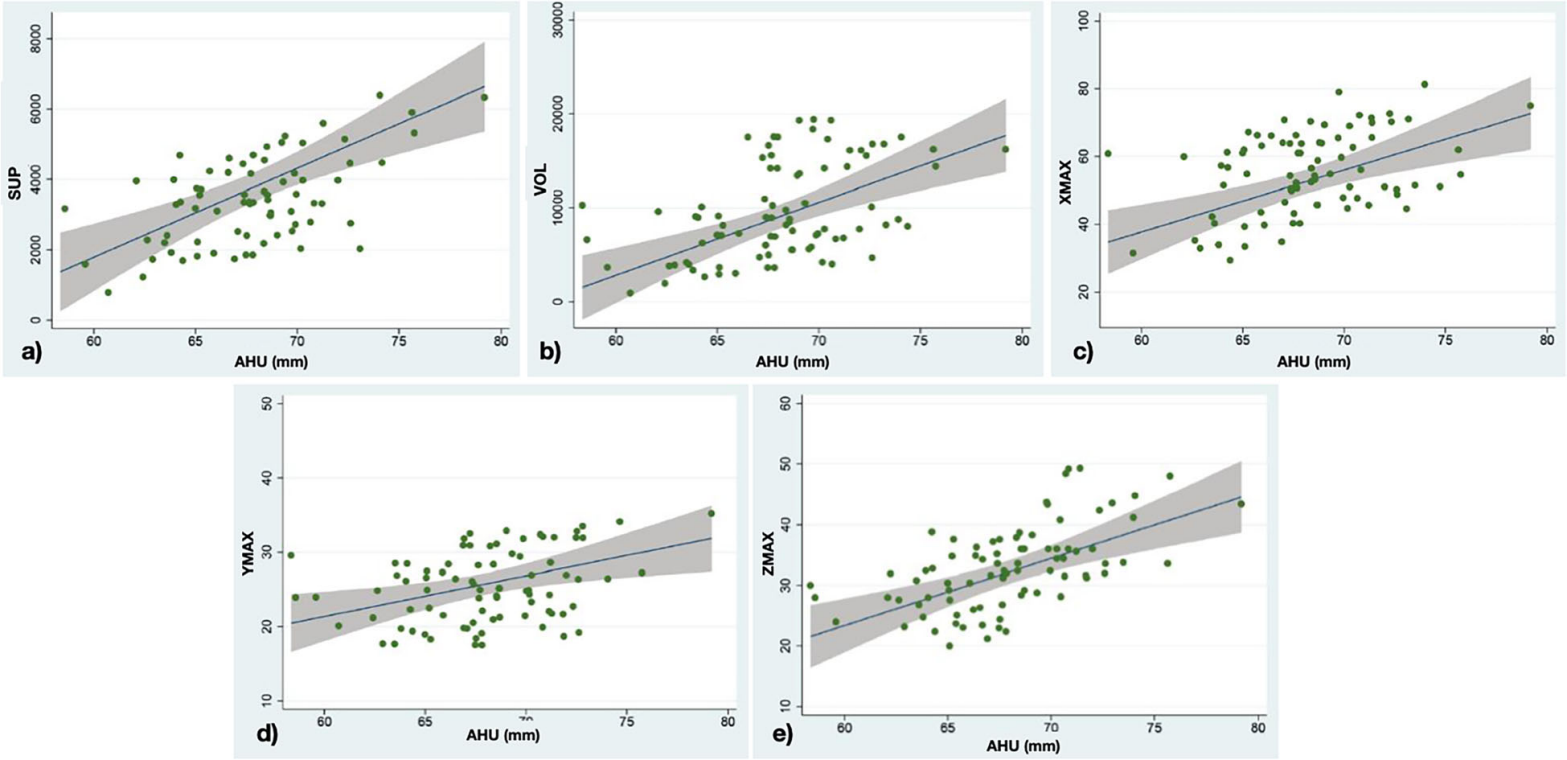
**A** Linear regression between the surface dimensions of the frontal sinus (sup), in the ordinate, and the vertical skeletal anterior superior dimension (AHU), in the abscissa. **B** Linear regression between the volume quantities of the frontal sinus (vol), in ordinate, and anterior superior skeletal vertical dimension (AHU), in abscissa. **C** Linear regression between the width of the frontal sinus (xmax), in ordinate, and anterior superior skeletal vertical dimension (AHU), in abscissa. **D** Linear regression between the depth of the frontal sinus (ymax), in the ordinate, and anterior superior skeletal vertical dimension (AHU), in the abscissa. **E** Linear regression between the height of the frontal sinus (zmax), in ordinate, and anterior superior skeletal vertical dimension (AHU), in abscissa

The univariate regression analysis showed that XMAX increases by 1.82 mm to the unit increase of the upper front vertical dimension; YMAX undergoes an increase of 1.9%; ZMAX of 1.1; the volume of 8.5%; the surface area of 6.6%. For all these values the confidence interval was found to be greater than 0, therefore statistical significance was achieved. Figure 5.

**Fig. 6:**
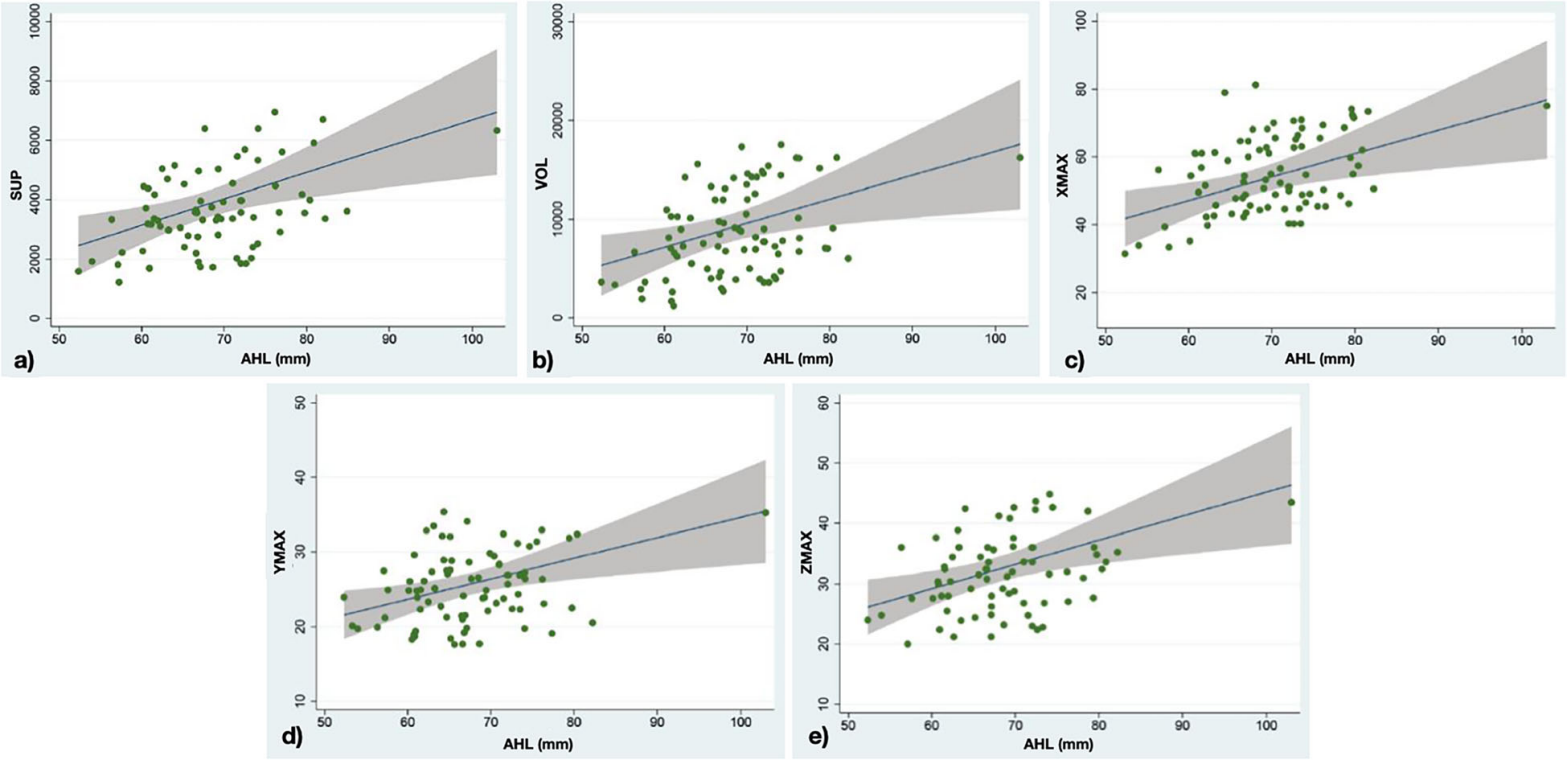
**A** Linear regression between the surface of the frontal sinus (sup), in the ordinate, and the lower anterior skeletal vertical dimension (AHL), in the abscissa. **B** Linear regression between the volume quantities of the frontal sinus (vol), in the ordinate, and the lower anterior skeletal vertical dimension (AHL, in the abscissa. **C** Linear regression between the width of the frontal sinus (xmax), in the ordinate, and the lower anterior skeletal vertical dimension (AHL), in the abscissa. **D** Linear regression between the depth of the frontal sinus (ymax), in the ordinate, and the lower anterior skeletal vertical dimension (AHL), in the abscissa. **E** Linear regression between the height of the frontal sinus (zmax), in the ordinate, and the lower anterior skeletal vertical dimension (AHL), in the abscissa

The univariate regression analysis for the lower anterior vertical dimension identifies a statistically significant relationship with all the five-dimensional parameters examined for the frontal sinus. Specifically, XMAX increases by 0.69 mm for each millimeter increment of lower DVA, YMAX by 1%, ZMAX by 0.4 mm, volume by 2.5%, surface area by 2.8% (Fig. 6).

Finally, the length of the skull base (S - N) is also related to the height, depth, width, volume and surface of the frontal sinus, in a statistically significant way. The increase of XMAX for each unit increase of the length S - N is 0.87 mm; YMAX increased by 1.94%; ZMAX of 0.6 mm; the volume of 6%; the surface of 4%.

From the analysis of multiple regression, also considering the age and sex of the patients, the values relating to the dimensional parameters of the frontal sinus and SNA, SNB, ANB and S - N no statistically significant correlation has been found. In contrast for the total DVA, the higher DVA, the lower DVA, adjusted for age and sex, the statistical significance was also confirmed by multiple regression analysis.

For the same sex and age:

- For each millimeter increment of total anterior skeletal vertical dimension, XMAX increases by 0.86 mm (CI= 0.43, 1.29), YMAX by 0.84% (CI= 0.1, 1.5), ZMAX by 0.47 (CI= 0.2, 0.7) mm, volume of 3.7% (CI= 1.6, 5.9), surface area of 3.1% (CI= 1.5, 4.7).
- For each millimeter increment of anterior superior skeletal vertical dimension, XMAX undergoes an increase of 1.62 mm (CI= 0.64, 2.71), ZMAX of 1.1 mm (CI= 0.49, 1.62), the volume of the 8,5% (CI= 1.40, 11.80) and surface area of 5.6% (CI= 1.8, 9.5).
- For each increase in lower anterior skeletal vertical dimension, XMAX increases by 0.56 mm (CI=0.04, 1.10), ZMAX by 0.33 mm (CI= 0.04, 0.61), volume by 2.5% (CI= 0.3, 0.5), the surface area of 2.2% (CI= 0.30, 4.10).

## Discussion

The maxillary sinus has a significant effect on diagnosis and semiology among craniofacial structures. Technological advances and the development of systems for the three-dimensional representation of this structure permit us to evaluate, through CBCT, specific alterations or sinus pathology. To date only few articles have been publish in literature assessing the relationship between frontal sinus and various cephalometric features using three-dimensional imaging. Gursoy et al. performed a recent research investigating the three-dimensional morphology of the frontal sinus in relation to the vertical dimension, but they assessed only linear measurement without calculating the volume and surface of the sinus [27].

Thus, the present study has investigated the correlation between the volume, surface, and maximum depth, height and width of the frontal sinuses and the facial growth pattern. The sample has been categorized by age because the physiological dimensional changes of the frontal sinus after 12 years of age are minimal and the sinuses growth rate after the growth peak slows down. For this reason, patients at the end of their pubertal growth spurt were considered.

The results of the 3D evaluation showed that volume, surface, and depth of the frontal sinus are on average greater in men than in women with statistically significance, confirming the results of a similar previous study [34].

The increasing of the volume, the surface, and the three dimensions of the frontal sinus recorded was correlated with the increasing of the age of the subjects with no statistically significance.

These two associations have already been investigated with smaller samples, on cephalograms in postero-anterior projects, therefore the bidimensional measurements were not realistic [35, 36].

In a retrospective study conducted on 32 skulls, the paranasal sinuses evaluation by CBCT showed that the depth and the volume of the frontal sinus increased with age (*p* <0.05) [37]. The sample of this study was divided into three age groups with a much greater variability (from 21 to 80 years) compared to ours.

Buyuk et al. [35] investigated the association between the frontal sinus morphology and the sex of 148 Turkish young adult subjects. The measurements were performed on bidimensional postero-anterior cephalograms and the authors found that the frontal sinus were larger in males than in females as demonstrated in our study. Moreover, the aforementioned study affirmed that frontal sinuses were unique due to their morphological characteristics to each individual, and in the field of forensic science, these features were very significant for personal identification.

In a further study, which aimed to investigate whether there was a relationship between skeletal maturation and frontal sinus morphology, through measurements made on postero-anterior projection teleradiographs of 220 growing subjects (aged between 8 and 18 years), a statistically significant difference was found between the width of the frontal sinus in men and women, in favor of the former [36]. These results agree with those obtained by the authors of the present study. The authors subsequently investigated the possible relationship between frontal sinus size and facial morphometric and bio typological characteristics.

Previous investigations have pointed out a relation between frontal sinus characteristics and the craniofacial growth pattern [9, 38].

A research made by Rossouw et al. [9] showed that there was a significant correlation between the area of the frontal sinus, calculated on bidimensional latero-cephalograms, and the maxillary and mandibular length, symphysis dimension, and condylar length, assuming that the increase in volume of the frontal sinuses can be considered as an additional predictor for mandibular growth. In our research, the correlations related to the skeletal class, the position of the maxilla and mandible with respect to the base were investigated. The univariate regression models corroborate the results of the previous study, in fact it found a statistically significant interaction between the SNB angle, that represent the dimension of the mandibular bone, and the volume, surface, width and height of the frontal sinus.

However, the results present in literature are in contrast. Dah-Jouonzo et al. [38] assessed the relation between the frontal sinuses volume and the maxilla-mandibular relationship in 95 subjects and evinced that the volume of the sinus is mainly affected by the changes in vertical dimensional than in the sagittal one. The research observed also that the average sinuses volume was larger in patients presenting class III malocclusion, followed by class II division 1, whereas subjects with class II division 2 malocclusion and class I were comparable [38]. This study took into consideration a sample like ours in terms of number and in this case the measurements were carried out on CBCT scans. In fact, the results partially match ours, since also in this study the relationship between the anterior vertical skeletal dimension and the volume of the frontal sinuses is evident. In this case, however, the sample was not divided into age groups and by sex.

Other authors in a 2017 study involving 144 subjects hypothesized that the frontal sinus pneumatization pattern would affect the skeletal growth pattern and could be used as a growth predictor [39]. Skull teleradiographs in postero-anterior and latero-lateral projection were used to measure the size of the frontal sinus. The results of this study showed an association between an increased size of the frontal sinus and a reduced inclination of the anterior skull base. In males, larger frontal sinus sizes were associated with increased anterior vertical dimension; in females, on the other hand, to an increase in the gonial angle [39].

A study published by Said et al. [40], examining the postero-anterior and lateral cephalograms of 1226 patients, demonstrated the correlation between cephalometric parameters such as the anterior skull base, the facial height, the inclination of the upper incisors and the size of the frontal sinus. Since these assumptions, the authors were able to affirm that the volume of the frontal sinus could be used as an indicator of a correct and harmonious anterior occlusion (22). The sample in this case is extremely large, however the limit of this study consists in the having carried out the measurements on two-dimensional radiographic examinations that are not able to return real measurements of the anatomical structures, but perspective. The results obtained are however in line with those of the current study.

A more recent investigations performed on lateral cephalograms by Yassaei et al. [41] demonstrated that paranasal sinus such (frontal and maxillary sinus) were bigger in patients presenting class III malocclusion than those presenting class 1 and class 2. Moreover, the authors showed a statistically significant positive correlation between the frontal sinus width and the dimension of the anterior cranial base. In contrast to the results of the present study, the previous study reported no statistically significant correlation has been found between the indexes of the skeletal vertical dimensions and the frontal sinus dimensions [41]. The reason behind this difference in results can be attributed to the use of lateral cephalometric radiographs. Overlap of the right and left frontal sinus on lateral cephalograms produce an inaccurate identification of the anatomical structures. In the present study, the three-dimensional frontal sinus measurements (volume, surface, height, width, and depth) were computed on the sagittal axial, and coronal sections of the CBCT scans, each sinus has been isolated and reconstructed with a 3D rendering which enabled more accurate measurements.

The diffusion of the CT cone beam in the dental field has allowed the volumetric study of the anatomical structures with high reliability. This study will allow us to have a clearer view of the mechanisms that regulate function and growth.

As previously demonstrated craniofacial alterations could frequently be related to the airway problems, so the necessity of assessing the correlation between frontal sinus morphologies and craniofacial features may be relevant. In 1993 Wolf et al. [42] reported that paranasal sinus growth was correlated with cranio-facial and dental growth. In this case our findings could provide an understanding of a possible relationship between frontal sinus development and facial growth.

Based on the present results, it can be said that young adults in whom the frontal sinuses have reached their maximum size (while vertical growth continues) a larger frontal sinus may be associated with future vertical growth. Longitudinal studies are required to state that the frontal sinuses can be considered as predictors of growth.

## Conclusions

By considering the results of the present study, the research shows that the volume, the surface and the depth of the frontal sinus are on average greater in men than in women, in a statistically significant way. For the width and height parameters, this difference can be found but with no significant difference.

Regarding the dimension of the frontal sinus correlated to age, although an increase in the volume, surface, and dimensions (x, y, z) of them was found, a statistical significance has not been reached for any of these parameters.

An inverse relationship emerged between ANB and the size of the frontal sinuses. A significant association was also found between depth, surface and volume of the frontal sinus and the protrusion of the mandible.

In addition, an increase in the size of the frontal sinus was recorded with both the total anterior vertical dimension and the upper and lower dimensions, taking into consideration the sample studied in its entirety. Also considering the sex and age of the patients, these values maintain statistical significance, confirming the correlation between the anterior vertical skeletal dimension and the dimensional characteristics of the frontal sinus of the subjects.

Similarly, the dimensional increase of the frontal sinus corresponds to an increase in the length of the skull base.

In conclusion, within the limitation of the present experimental study, it allows us to state that, despite the inter-individual variability of the frontal sinuses, it is possible to correlate some craniofacial bio typological aspects.

## Supplementary information

**Additional file 1**

## Data Availability

The datasets used and/or analyzed during the current study are available from the corresponding author on reasonable request.
